# Eosinophilic mastitis: a rare benign inflammatory condition and review of the literature

**DOI:** 10.1093/jscr/rjac456

**Published:** 2022-10-12

**Authors:** William Arnott, Gregory Leong, Arie Davis, Jason Diab, Zackariah Clement

**Affiliations:** The Tweed Hospital, Department of General Surgery, Tweed Heads, New South Wales, Australia; John Flynn Private Hospital, Departmemnt of General Surgery, Tugun, Queensland, Australia; University of Notre Dame, School of Medicine, Darlinghurst, NSW, Australia; The Tweed Hospital, Department of General Surgery, Tweed Heads, New South Wales, Australia; Bond University, School of Medicine, Robina, Queensland, Australia; The Tweed Hospital, Department of General Surgery, Tweed Heads, New South Wales, Australia; University of New South Wales, School of Medicine, Kensington, New South Wales, Australia; The Tweed Hospital, Department of General Surgery, Tweed Heads, New South Wales, Australia; John Flynn Private Hospital, Departmemnt of General Surgery, Tugun, Queensland, Australia; University of Notre Dame, School of Medicine, Darlinghurst, NSW, Australia; University of New South Wales, School of Medicine, Kensington, New South Wales, Australia; The Tweed Hospital, Department of General Surgery, Tweed Heads, New South Wales, Australia; John Flynn Private Hospital, Departmemnt of General Surgery, Tugun, Queensland, Australia

## Abstract

We report the case of a 53-year-old nulliparous female presenting with a 9-month history of recurrent mastitis and a retro-areolar lesion. Histological assessment showed an inflammatory infiltrate predominantly composed of eosinophils without evidence of malignant changes. The patient was diagnosed with eosinophilic mastitis and commenced on a course of oral steroids with good effect. This case will outline the pathology, clinical manifestations and diagnosis of eosinophilic mastitis alongside a review of the literature.

## INTRODUCTION

Eosinophilic mastitis is a rare benign inflammatory breast condition characterized by the infiltration of eosinophils in breast tissue. The subsequent activation and degranulation of eosinophils lead to release of cytotoxic inflammatory proteins often resulting in the formation of an inflammatory mass accompanied by erythema and tenderness. The literature reports more frequent presentations of eosinophilic infiltrate in the lungs, liver, skin and the gastrointestinal tract [1–3]. The breast is an uncommon site of eosinophilic infiltration with very few cases reported (Table 1). It can sometimes present with similar features as malignant breast cancer on clinical and radiological assessment posing diagnostic challenges for clinicians.

**Table 1:** A literature review of case reports on eosinophilic mastitis

Author	Year	Patient presentation	Imaging	Serology	Histology	Management	Results
Wilsher and Banerjee [9]	2020	Thirty-five-year-old non-asthmatic female 4-month post-partum and breast feeding, with a palpable non-tender, mobile mass in upper outer quadrant of right breast.	**Ultrasound**: 1.2 cm cystic solid ovoid lesion, with moderate vascularity. No overlying skin thickening or inflammatory change (BI-RADS 3).	Nil serum eosinophil count recorded.	**Core needle biopsy**: predominant eosinophilic inflammatory infiltrate, with associated histiocytes and plasma cells within the stroma. Accompanied by lobularity and ductal dilatation.	Nil active treatment administered.	Reduction in size of lesion on ultrasound after 3 months. Normal eosinophil count after 5 months. Persistence of small palpable mass at 9-month follow-up.
Bang *et al* [26]	2021	Forty-three-year-old asthmatic, ex-smoking female, presenting with unilateral breast swelling and erythema.	**Ultrasound**: hyperechoic areas, oedematous change, dilated lymphatics and increased vascularity (BI-RADS 4C). Axillary level 1 lymphadenopathy. **CT-chest + MRI**: extensive fibro-glandular and oedematous changes with increased vascularity, but no mass enhancement. Axillary lymphadenopathy.	Leucocytosis with an elevated eosinophil count and differential.	**Core needle biopsy: breast:** eosinophilic infiltration of lympho-plasma cells and reactive hyperplasia. **Axillary lymph node**: reactive hyperplasia with eosinophils.	Oral steroids at 60 mg/day along with antibiotics and leukotriene receptor antagonist for 14 days. Steroids then titrated to 5 mg/day over the next 5 months.	Reduction in swelling and erythema at 2 weeks. Serum eosinophil count gradually returned to normal after 4 months. Ultrasound at 12-month follow-up showed nil abnormalities in the affected breast.
Bajad *et al* [17]	2019	Twenty-seven-year-old asthmatic non-smoking female with a history of urticarial skin rashes, presenting with a painful mobile mass in the right breast accompanied by nipple discharge.	**Ultrasound**: thickening of multiple ducts with surrounding architectural distortion and hyperreflexive parenchyma.	Normal leukocyte count with elevated absolute eosinophil count and differential.	**Core needle biopsy**: dense periductal and lobular mixed inflammatory infiltration composed of sheets of eosinophils.	Oral steroids and antihistamines (duration and dose not disclosed).	Complete resolution at 6 weeks follow-up ultrasound.
Takahashi [27]	2018	Thirty-six-year-old asthmatic female, presenting with a painful left breast mass.	**Ultrasound**: heterogeneous mass with unclear borders with a spotty vascular pattern. **Mammography**: nodular shadow with asymmetry. **MRI**: poorly defined enhancement without distinct tumour formation.	Elevated eosinophil count.	**Core needle biopsy**: inflammatory fibrosis with diffuse infiltration of eosinophils and lymphocytes.	Anti-allergic agents (duration and dose not disclosed).	Gradual reduction in pain, induration and serum eosinophil levels over 4 months. No symptom recurrence at 3-year follow-up.
Parakh *et al*. [18]	2021	Fifty-one-year-old postmenopausal diabetic female with no history of asthma or atopy, presenting with a painful erythematous right periareolar region and an ill-defined lump.	**Ultrasound**: hyperreflective parenchyma with skin thickening, prominent dilated ducts with wall thickening. **Mammography**: increased trabecular density with prominent lymph nodes (BI-RADS 1). **MRI**: heterogeneously dense, oedematous fibro-glandular tissue with non-mass enhancement. Mild prominence of ducts with dilatation. Axillary lymphadenopathy.	Normal absolute eosinophil count.	**Core needle biopsy**: dense periductal inflammatory infiltrate comprising predominantly of eosinophils.	Treatment initially with antibiotics. Later treatment changed to steroids following histological diagnosis (duration and dose not disclosed).	Slight reduction in erythema with antibiotics. Presently awaiting review following commencement of steroid therapy.
Singh *et al* [16]	2015	Thirty-year-old female with a history of allergic rhinitis, presenting with bilateral mastalgia and mobile bilateral breast lumps associated with nipple discharge.	Ultrasound: bilateral ill-defined heterogenous hypoechoic areas. Unorganized phlegmon formation and oedema in subcutaneous layer. Bilateral lymphadenopathy. Mammography: widespread inflammatory changes. No discreet mass lesions visualized.	Leucocytosis with raised absolute eosinophil count and differential.	Fine needle aspiration: inflammatory infiltrate composed mainly of eosinophils on a necrotic background. **Surgical incisional biopsy**: massive infiltration of eosinophilic granulocytes around ducts and lobules filled with intraluminal secretory material with reactive epithelial changes.	Antibiotics (duration and dose not disclosed). Followed by bilateral incision and drainage of two lesions.	No response to antibiotic therapy.
Komenka *et al* [4]	2003	Fifty-year-old asthmatic female with a history of superficial thrombophlebitis presenting with: Mass 1 (Feb-1999): painless mobile right breast mass at the central-lateral 9 o’clock position. Mass 2 (Aug-2002): recurrence of breast non-tender, firm mass to right breast at the lower outer quadrant.	Mass 1: Ultrasound: 4.5 cm solid lesion. Mammography: well circumscribed nodular density Mass 2: Ultrasound: complex 6 × 3.7 cm mass. Mammography: indeterminate round nodule	Normal total leucocyte count, with a raised eosinophil differential.	Mass 1: Fine needle aspirate: inconclusive inflammatory changes. **Complete surgical excisional biopsy**: inflammatory infiltrate composed predominantly of eosinophils. Reactive hyperplasia of ducts and lobules. Mass 2: **Complete surgical excisional biopsy**: diagnostic for eosinophilic mastitis.	**Mass 1:** Complete surgical excision with clear margins. Oral montelukast and cromolyn sodium (duration and dose not disclosed). **Mass 2:** Complete surgical excision with clear margins.	Persistence of serum eosinophilia and recurrence of breast mass despite treatment with montelukast and cromolyn sodium and complete surgical excision with clear margins of Mass 1.
Garg *et al* [28]	2001	Fifty-year-old female – subsequently diagnosed with asthma – presenting with painless enlarging breast lump and ipsilateral axillary lymphadenopathy.	**Mammography**: large ill-defined hetero-dense opacity in the retro-areolar region without microcalcification.	Raised leucocyte count with an elevated absolute eosinophil count and differential.	**Core needle biopsy**: moderate mixed acute on chronic inflammation and periductal fibrosis. **Surgical excision biopsy**: diffuse and dense periductal and stromal inflammatory infiltrate comprising sheets of eosinophils.	Oral steroids (duration and dose not disclosed).	Excellent response to treatment at 15-day follow-up, and complete resolution of breast lesion documented at 2-month review.
Bocal Topal *et al*. [29]	2007	Sixty-year-old female with asthmatic bronchitis, presenting with a tender upper outer quadrant and subareolar region of right breast.	**Ultrasound**: a 15 × 7 mm heterogenous mass. Enlarged ipsilateral axillary lymph node with cortical thickening. **Mammography**: asymmetric density with no mass or microcalcification (BI-RADS 4).	Raised eosinophil differential.	**Core needle biopsy**: inflammatory process with extensive ductal cellularity rich in eosinophils.	Oral corticosteroids (duration and dose not disclosed).	Excellent response to treatment, with complete resolution of ultrasonographic and mammographic findings at unspecified interval review.

## CASE REPORT

A 53-year-old female was referred by her general practitioner to a breast surgeon following three recurrent episodes of unilateral mastitis over 9 months involving the left lower inner quadrant and nipple-areolar complex (NAC). Her medical history included uterine fibroids and menorrhagia, which was managed with an intrauterine device and norethisterone. She had no significant surgical history and no family history of breast or ovarian cancer. She denied any allergies or atopic sensitivities and was a non-smoker. She had previously undergone eight cycles of *in vitro* fertilization but remained nulliparous.

**Figure 1:**
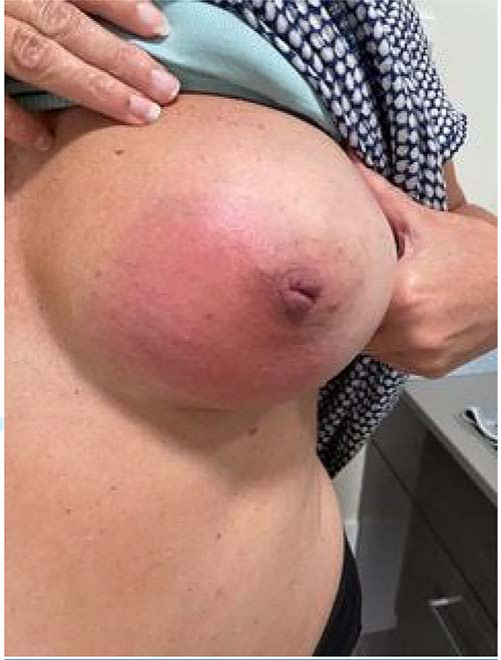
Photo of patients left breast.

On initial examination in April 2021 by the breast surgeon, the left lower inner quadrant and NAC of the left breast were pigmented with thickened skin (Fig. 1). The nipple was inflamed and cracked with a firm underlying mass without nipple discharge. There were no associated systemic features. The contralateral breast was normal. There was no lymphadenopathy. The patient had a normal leucocyte count of 5.6 × 10^9^ with a normal absolute eosinophil count of 0.38 × 10^9^.

**Figure 2:**
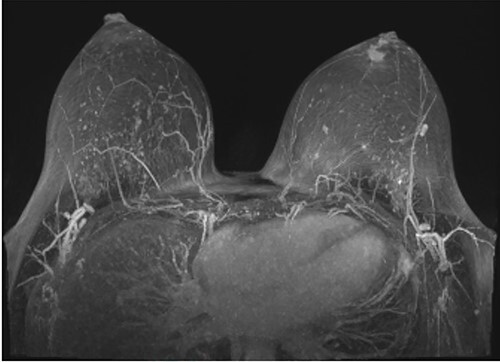
MRI of breasts showing left retro-areolar lesion.

**Figure 3:**
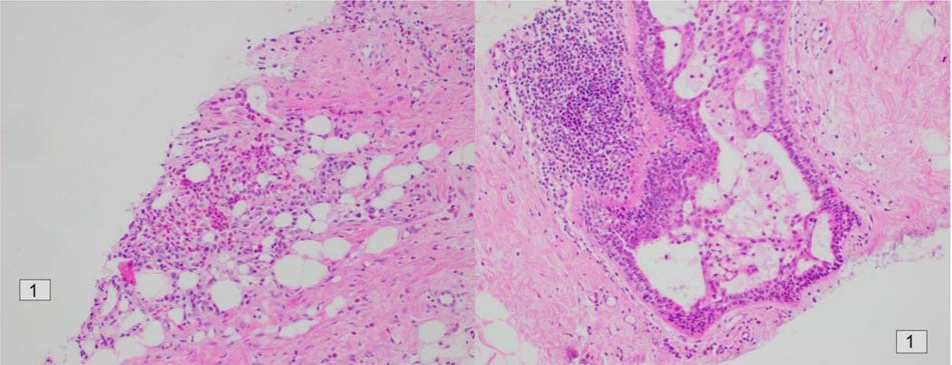
Histology of the patient’s left retro-areolar lesion.

**Figure 4:**
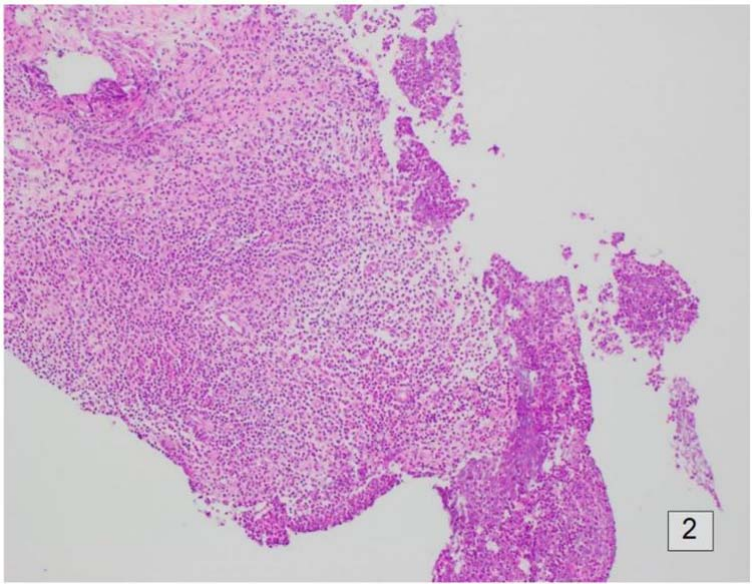
Histology of the surrounding breast tissue adjacent to the left retro-areolar complex.

Following initial review, the patient was referred for a magnetic resonance image (MRI) which demonstrated an indeterminate retro-areolar lesion in the left breast (Fig. 2). A targeted ultrasound and core needle biopsy also showed active chronic periductal, interlobular and interstitial inflammation with focal duct ectasia consistent with Breast Imaging Reporting and Database System (BI-RADS) score of 2. Histological analysis showed predominant eosinophilic infiltrate (Fig. 3). There was no evidence of granulomatous inflammation or malignancy and staining for fungal elements and mycobacterium was also negative. A biopsy of the adjacent skin showed ongoing periductal and interstitial chronic inflammatory cell infiltrate with focal abscess formation (Fig. 4).

Following biopsy, ciprofloxacin 500 mg was commenced for 6 weeks with sound improvement. Six months later, the patient re-presented to the breast surgeon with pain and swelling accompanied by erythematous changes overlying the same distribution of her left breast. After consultation with an infectious disease specialist, a trial of oral prednisolone was started at a dose of 25 mg per day for 5 days, tapered to 2.5 mg per day over the following 2 weeks. There was an excellent response to therapy with complete clinical resolution observed at 4 weeks follow-up. At time of publication, there has been no symptom recurrence.

## DISCUSSION

Eosinophilic mastitis is a benign inflammatory condition classically presenting with a unilateral breast mass, erythema and/or pain. It is diagnosed when eosinophilic infiltration is the predominant histological feature in affected breast tissue in the absence of fungal or parasitic infection. The eosinophilic infiltration and inflammatory response are thought to relate to an immune or allergic reaction to ductal secretions [4]. The clinical presentation poses a diagnostic dilemma to clinicians as it shares many clinical and radiological features with malignant breast disease, and thus the diagnostic focus is centred on histological evaluation of biopsied tissue.

Eosinophils are pleiotropic multifunctional leucocytes responsible for releasing cytotoxic granule proteins that play a role in combating infectious agents; they are important mediators of the allergic response along with basophils and mast cells [5]. Eosinophils comprise only a small proportion of total leukocytes in peripheral blood. However, in rare circumstances, the body fails to regulate the production of eosinophils and when there is an excessive accumulation of eosinophils, this condition is termed ‘eosinophilia’. When heightened levels of eosinophils exist in peripheral blood, the likelihood of infiltrate tissue activating cytotoxic and inflammatory responses increases.

Infiltration of eosinophils into breast tissue is rare. When present, it is often related to conditions such as idiopathic granulomatous mastitis (IGM) [6, 7], secondary to infection [8–10] or associated with systemic vascular disease such as Eosinophilic Granulomatosis with Polyangiitis (Churg-Strauss syndrome) [11–13]. In these conditions, the eosinophilic infiltration is usually accompanied by lymphocytes, epithelial histocytes or Langerhan’s giant cells on a neutrophilic background, and often self-limited to the lobules of the breast rather than periductal [6, 14, 15]. In the rare case of eosinophilic mastitis, eosinophils comprise the main infiltrate in sampled inflammatory tissue and there is an absence of an alternative inflammatory process such as granulomatous inflammation, vasculitis or infection.

The literature reports the most common clinical features of eosinophilic mastitis include a mobile breast lump often associated with pain, erythema and skin thickening (Table 1). However, two cases also reported nipple discharge [16, 17]. Serum eosinophilia was present in all but one case [18], whereas only two cases of the disease were reported in patients without a history of asthma or allergic rhinitis [9, 18].

First-line investigations should include an ultrasound, mammography and an MRI. Ultrasound findings commonly showed a solid or heterogenous mass, skin thickening and duct dilatation with associated wall thickening. There was often increased vascularity related to these tissue changes. Mammography frequently demonstrated an asymmetrical density within the affected breast, whereas MRI commonly showed oedematous changes but not always a mass or tumour. One MRI study did identify duct dilatation [18].

It remains difficult to clinically and radiologically differentiate eosinophilic mastitis from other benign and malignant breast conditions. The presence of erythema, skin changes and pain on clinical examination may be present in conditions such as granulomatous, infective or lactational mastitis. On mammography and ultrasound, granulomatous mastitis commonly shows asymmetry, increased tissue density, hypoechoic mass lesions or nodular structures of diffuse distribution [19]. Given eosinophilic mastitis commonly presents with a breast mass, initial examination should raise the suspicion for malignant disease. However, radiologically, the localized vascular, lymphatic, ductal and tissue changes seen on ultrasound in eosinophilic mastitis may mimic the changes seen in breast carcinoma [20].

Therefore, tissue diagnosis remains necessary to exclude malignancy and diagnose eosinophilic mastitis. Rarely is prominent eosinophilic infiltrate associated with malignant breast disease [7]. Core needle biopsy is the preferred tissue sampling modality – rather than fine needle aspiration – due to the fibrous nature of inflammatory breast tissue [21, 22]. One case reported a missed eosinophilic mastitis on initial fine needle aspirate [16]. Histologically, eosinophilic mastitis is characterized by periductal, interlobular and interstitial infiltration by eosinophils often with ductal dilatation and accompanying fibrosis and epithelial cell atypia or reactive hyperplasia [17].

The underlying pathogenesis of eosinophilic mastitis is not well understood. Recent studies have hypothesized that eosinophilic infiltration of the breast may be associated with localized allergy to intraluminal substances [4, 7]. Likewise, the pathogenesis of eosinophilic gastroenteritis is thought to represent a localized reaction to allergens within the gastrointestinal tract [1, 23, 24]. A similar hypersensitivity response of the breast ducts and lobules may account for our patient’s underling inflammatory breast changes and eosinophilic infiltration, especially in the absence of infective aetiology [16].

The mainstay of treatment for eosinophilic mastitis remains steroid therapy with serial reviews [25]. In Bang’s case study, successful resolution was achieved with a daily dose of 60 mg of prednisolone, titrated over 5 months [26]. Comparatively, effective treatment with anti-histamines alone has been reported [27], as well as in conjunction with steroid therapy [17]. Our approach utilized a judicious use of systemic steroids with an initial dose of 25 mg of prednisolone per day for a period of just 5 days, titrating the dose over a further 7 days. The literature confirms our belief that surgery has a limited role in resolution of symptoms (Table 1) [4, 16].

The case highlights the importance of a systematic multi-disciplinary approach in managing disease processes with benign and malignant features. In our case, histological evaluation of affected breast tissue showed predominant eosinophilic infiltrate, representative of eosinophilic mastitis and non-surgical treatment with corticosteroids was effective in resolving the patient’s symptoms. Our case shows that the absence of eosinophilia in serum testing cannot alone exclude eosinophilic mastitis, which is similarly exhibited in Parakh’s case report [18]. This case demonstrates the challenge in diagnosis and treatment of inflammatory breast conditions, as the non-specific clinical signs can masquerade as other conditions and lead to ineffective or even unnecessary invasive management.

## CONCLUSION

Eosinophilic mastitis is a localized benign inflammatory response isolated to breast tissue associated with significant infiltration of eosinophilic granulocytes around ducts and lobules. This may be secondary to intraluminal secretary material with reactive epithelial changes in the absence of microbial growth. Although the disease shares many clinical and radiological similarities with breast carcinoma, the mainstay of treatment is non-surgical, and conservative steroid therapy is considered an appropriate first-line approach. With a sound understanding of the benign nature of the disease, a systematic approach can be delivered without the need for surgical intervention.

## CONFLICT OF INTEREST STATEMENT

No authors have any competing interests. Patient consent has been obtained from patients in line with ethical practice and guidelines. There is no source of financial or other support and no financial or professional relationships which may pose a competing interest. The data are deemed confidential and under ethics cannot be disseminated openly due to confidentiality and privacy.

## CONTRIBUTORS

The authors contributed to the conception and design of the manuscript, revised it critically for important intellectual content, approved the final version to be published and agreed to be accountable for all aspects of the work.
